# Prevalence of alpha-synuclein seeding activity in the Olfactory Mucosa following SARS-CoV-2 infection: results from a pilot study

**DOI:** 10.1007/s00401-026-03104-4

**Published:** 2026-10-22

**Authors:** Beatrice Heim, Klaus Seppi, Nicolas De Cleene, Matilde Bongianni, Erika Bronzato, Clancy Cerejo, Frank Jagusch, Philipp Kindl, Judith Löffler-Ragg, Günter Weiss, Timo Gottfried, Joachim Schmutzhard, Gianluigi Zanusso, Werner Poewe

**Affiliations:** 1 Department of Neurology, Medical University of Innsbruck, Anichstrasse 35, 6020 Innsbruck, Austria; 2 Department of Neurosciences, Biomedicine and Movement Sciences, University of Verona, Verona, Italy; 3 Department of Internal Medicine II, Medical University of Innsbruck, Innsbruck, Austria; 4 Department of Otorhinolaryngology, Medical University of Innsbruck, Innsbruck, Austria

**Keywords:** COVID-19, Olfaction, Olfactory mucosa, α-synuclein, RT-QuIC, Parkinson’s disease, Hyposmia

## Abstract

Olfactory dysfunction (OD) is a hallmark of SARS-CoV-2-infection where it is believed to result from neuroinflammation induced by viral invasion via the olfactory mucosa (OM). Hyposmia also affects the majority of patients with Parkinson’s disease (PD) where it is caused by α-synuclein (aSyn) pathology in the olfactory system, and aSyn-seeding-activity can be detected in the OM using aSyn-seeding-amplification-assays (aSyn-SAA). The present pilot study aimed to investigate whether SARS-CoV-2-infection induces local aSyn-misfolding and -aggregation in OM samples using aSyn-SAA. We conducted an observational study including PD patients (n = 51), individuals with a history of documented symptomatic SARS-CoV-2-infection (post-COVID; n = 95), and healthy controls (HC; n = 42). Post-COVID-participants were stratified based on persistent OD (COVID+OD; n = 42) or normal olfaction (COVID–OD; n = 53) using Sniffin’-Sticks-Identification (SSI) and -Discrimination (SSD) tests. OM samples were obtained by ENT specialists and analyzed by aSyn-SAA at the University of Verona. Clinical phenotyping included MDS-UPDRS, MoCA, and the Innsbruck-RBD-Inventory. OM-SAA positivity rates were significantly different between PD and both post-COVID-participants and HCs (80.4% vs 24.2% vs 11.9%; *p* < 0.001). Within the post-COVID-group, positivity rates were higher in COVID+OD vs COVID–OD (35.7% vs 15.1%; *p* = 0.030), and COVID+OD differed from HC (*p* = 0.030), while COVID–OD did not (*p* = 0.65). Among post-COVID-participants, aSyn-SAA positivity was associated with lower SSI (*p* = 0.007) and SSD (*p* < 0.001) scores, but not with MDS-UPDRS, MoCA, or RBD measures. OM-SAA-positivity was enriched among individuals with persistent post-COVID-OD and correlated with olfactory impairment. These findings support the hypothesis that neuroinflammation following SARS-COV-2-infection might promote local aSyn-aggregation. The biological and prognostic relevance of OM-SAA positivity is unknown and needs further prospective investigation.

## Introduction

Loss of smell is among the most frequent and distinctive symptoms of SARS-CoV-2 infection. Hyposmia is also a characteristic non-motor feature of Parkinson’s Disease (PD) which often precedes the onset of motor symptoms by years or decades, and it has been suggested that α-synuclein (aSyn) pathology in PD may start in the olfactory system and spread via cell-to-cell transmission along interconnected neural circuits [7, 8, 13, 14, 20, 23, 29].

SARS-CoV-2 may reach the nervous system via olfactory routes, and experimental data indicate that viral neuroinvasion and inflammation can promote aSyn misfolding [2, 12, 16, 39]. This has raised concerns about a potential of SARS-CoV-2 infections to induce PD and several case reports were published suggesting such a mechanism [9].

Recent studies have reported aSyn seeding activity using seeding amplification assays (SAAs) on olfactory mucosa (OM) samples in subjects with PD and Dementia with Lewy Bodies (DLB) and also in individuals with isolated REM sleep behaviour Disorder (iRBD) who are at risk for either of these diseases [5, 36]. This pilot study was designed to compare the rates of positive aSyn seeding activity as assessed by SAA in OM samples (OM-SAA) from individuals with a recent history of confirmed COVID-19 and healthy controls without a history of SARS-Cov-2 infection. In addition, a group of patients with clinically established PD were studied as positive controls. We further examined associations between OM-SAA status, olfactory performance, parkinsonian features, cognitive function, and a history of REM sleep behavior disorder (RBD).

## Methods

### Study design and participants

This observational pilot study (ClinicalTrials.gov: NCT05401773) included four groups: patients with Parkinson’s disease (PD) as positive controls, individuals with prior SARS-CoV-2 infection and persistent olfactory dysfunction (COVID+OD), individuals with prior SARS-CoV-2 infection without olfactory dysfunction (COVID−OD), and healthy controls (HC).

Potentially eligible participants were identified through a broad screening approach, including consecutive referrals from the specialised post-COVID outpatient clinic as well as participants from a previous observational studies conducted at our centre [28, 31], and personal contacts or word of mouth among hospital staff and their relatives. PD participants were recruited from the movement disorders outpatient clinic.

All participants were aged ≥18 years and able to understand the study procedures. Both COVID groups required documented, PCR-confirmed SARS-CoV-2 infection ≥ 3 months before inclusion. COVID+OD required olfactory dysfunction persisting for ≥ 3 months after infection, scores < 13/16 on both the Sniffin’ Sticks Identification (SSI) and Discrimination (SSD) subtests, and no structural nasal pathology potentially explaining the dysfunction. OD was attributed to SARS-CoV-2 based on its onset in temporal association with infection. COVID−OD required no history of or current OD and scores ≥ 13/16 on both subtests. Both COVID groups excluded participants with OD predating infection or relevant pre-existing neurological disorders. HC had no history of COVID-19, OD, or relevant neurological disorders, subjectively normal olfaction, and SSI and SSD scores ≥ 13/16. PD participants met established clinical diagnostic criteria [27], had no history of COVID-19, and served as positive controls for the RT-QuIC assay. All groups required a negative SARS-CoV-2 antigen test at inclusion; HC and PD participants additionally required a negative SARS-CoV-2 antibody test unless vaccinated (ClinicalTrials.gov: NCT05401773).

The study was approved by the local ethics committee in March 2021, and recruitment continued until January 2024. All participants provided written informed consent before participation. Post-COVID-19 participants were followed for at least one year, as described previously [28, 31].

### Clinical assessments

All participants underwent a standardized neurological examination, including an assessment of non-motor symptoms of PD, performed by study investigators (BH, KS, NDC, FJ, CC) under the supervision of trained movement-disorder specialists (BH, KS). Motor and non-motor symptoms were assessed using the Movement Disorder Society Unified Parkinson’s Disease Rating Scale (MDS-UPDRS, Parts I–III). In addition, cognitive function was screened using the Montreal Cognitive Assessment (MoCA) [22], and the Innsbruck REM Sleep Behavior Disorder Inventory (RBD-I) was used to identify symptoms suggestive of RBD, with a normalized score of ≥ 0.25, corresponding to at least two positive responses, being defined as a positive screen [11]. Olfactory function was assessed using the Identification (SSI) and Discrimination (SSD) subtests of Sniffin’ Sticks test battery (Burghart Medizintechnik, Wedel, Germany). To minimize cross-infection risk during the COVID-19 pandemic, odors were presented using a single-use paper sheet to which the tip of the reusable odor pen had been applied, in accordance with the recommendations of the German Society of Otorhinolaryngology, Head and Neck Surgery; consequently, the threshold subtest was omitted because it could not be safely administered under these conditions [15].

### OM sampling and RT-QuIC

Olfactory mucosa (OM) samples were collected by ENT specialists medial of the middle nasal turbinate in the direction of the superior nasal turbinate using FLOQ-Brushes (Copan Italia) [4]. Following the OM collection, swabs were stored at 4 °C, and shipped on dry ice to the University of Verona for processing and aSyn-SAA analysis, as previously published [5, 36].

In particular, recombinant full-length human α-synuclein (aSyn; amino acids 1–140) was prepared in-house. aSyn cDNA was cloned into pET-28a and expressed in *E. coli* BL21 (DE3). After induction with 0.1 mM IPTG, the periplasmic fraction was isolated, subjected to ammonium sulfate precipitation, and further purified via anion-exchange chromatography (Q-Sepharose, GE Healthcare). Protein purity was verified by SDS-PAGE, and aliquots were dialyzed into 10 mM sodium phosphate buffer (pH 7.4) and stored at − 80 °C.

For aSyn-SAA, 2 µL of diluted OM homogenate was incubated in 98 µL reaction buffer (100 mM phosphate buffer, pH 8.2; 10 µM Thioflavin T; 0.1 mg/mL recombinant aSyn; 0.004% SDS) containing ~ 37 mg of 0.5 mm glass beads. Reactions were run in triplicate at 30 °C in a FLUOstar® Omega plate reader under cycles of shaking (1 min at 200 rpm, double orbital) and rest (14 min), with fluorescence recorded every 45 min (excitation 450 ± 10 nm, emission 480 ± 10 nm).

A reaction was considered positive if fluorescence exceeded 10% of the plate’s maximum signal. OM samples were classified as positive if ≥ 2 of 3 replicates crossed this threshold within 50 h, negative if none did, and inconclusive if one of three was positive. Inconclusive samples were reanalyzed and, if results persisted, classified as negative [5].

### Statistics

All statistical analyses were performed using IBM SPSS Statistics (version 30.0; IBM Corp., Armonk, NY, USA). Age, sex and OM-SAA results were compared among people with PD, individuals with post-COVID-19, and HC. Group comparisons were performed using chi-square tests with Benjamini–Hochberg FDR-corrected post-hoc analyses, and a univariate ANOVA followed by Šidák-corrected post-hoc tests. As the PD cohort was included as a positive control group for the OM-SAA, the PD cohort was not included for further group comparisons.

Chi-square tests were applied to assess differences in categorical variables (e.g., sex, persistent hyposmia, OM-SAA positivity) among COVID+OD, COVID-OD, and HC. Group differences in OM-SAA positivity were analyzed using contingency-table statistics. Counts of OM-SAA–positive and –negative individuals were cross-tabulated across three groups (COVID+OD, COVID-OD, and HC). The overall association between group and OM-SAA status was assessed using a two-sided chi-square test of independence (2×3 design), with post-hoc comparisons corrected using the Benjamini–Hochberg FDR procedure; expected cell counts satisfied assumptions for χ^2^ approximation. Effect size was reported as Cramer’s V. To complement these analyses, column-proportion tests with z-statistic annotation were applied for descriptive pairwise comparisons, where groups sharing the same subset label did not differ significantly at *p* < 0.05. To account for age differences across groups, we conducted a multivariable logistic regression predicting OM-SAA positivity, entering age and sex as covariates. Model significance was assessed using the omnibus χ^2^ statistic, and effects are expressed as odds ratios (95% CI). Additionally, OM-SAA positivity was modelled using multivariable logistic regression for those contrasts identified as significant in the chi-square analysis, with age and sex included as covariates and *p* values adjusted via Benjamini–Hochberg FDR correction.

Continuous outcomes (e.g., MoCA and MDS-UPDRS scores) were analyzed using analysis of covariance (ANCOVA) models controlling for age and sex, with post-hoc comparisons corrected using the Šidák procedure. Within the post-COVID cohort, differences between participants with and without positive OM-SAA results, were assessed for key demographic and clinical measures. To account for potential confounding by age and sex, analysis of covariance (ANCOVA) model was applied to these continuous outcomes.

Age and time since COVID-19 are reported as mean ± standard deviation (SD). All remaining continuous variables are summarized as mean ± standard deviation (median; 95% confidence interval of the mean), while categorical variables are presented as absolute and relative frequencies.

Due to the lack of existing prevalence data regarding OM-SAA of COVID-19 patients and significant uncertainty regarding their prevalence in HC, a formal power calculation was not conducted. The study was designed as an exploratory pilot to inform future research. The significance threshold was set at *p* < 0.05 (two-sided, unless stated otherwise). Post hoc pairwise comparisons were performed and reported only when the corresponding overall test was statistically significant. For multiple comparisons, Benjamini–Hochberg FDR correction was applied to chi-square and multivariable logistic regression analyses, and the Šidák procedure was used for ANOVA and ANCOVA, with all *p* values referring to the adjusted level unless otherwise stated. *p* values are reported to two decimal places, except for values  < 0.05, which are reported to three decimal places or as *p* < 0.001.

Because participants were recruited consecutively, age was not fully balanced across the HC and post-COVID-19 cohorts. Therefore, an age- and sex-balanced sensitivity analysis was performed. Equal-sized subsets were selected from each study group without replacement using a reproducible optimization procedure implemented in Python (version 3.12; Python Software Foundation, Wilmington, DE, USA), following the general principles of covariate balance in observational studies [37]. The optimization prioritized minimizing between-group differences in mean age, followed by balancing sex distribution, while maximizing the number of retained participants. The final optimized cohort comprised 33 participants per group. Analyses of the clinical outcome variables, including the MoCA and MDS-UPDRS scores, were repeated in this cohort to assess the robustness of the findings.

As an additional assessment of the robustness of the selection procedure, propensity scores for the probability of belonging to the COVID+OD group were estimated from age and sex using the Propensity Score Matching procedure implemented in IBM SPSS Statistics (version 30.0; IBM Corp., Armonk, NY, USA). The COVID+OD group was selected as the reference because it was the only group that differed significantly in age from both the HC and COVID−OD groups. Participants were ranked according to their estimated propensity scores, and a propensity score-based trimming procedure was applied to obtain equally sized, age-balanced groups. The resulting subset was highly consistent with the optimized sensitivity cohort, differing by only two participants in the COVID−OD group.

## Results

A total of 188 participants were included in the analysis: 51 PD patients, 95 post-COVID-19 participants (COVID+OD: n = 42, and COVID–OD: n = 53), and 42 HC. Table 1 summarizes demographic and OM-SAA results for all study groups. OM-SAA was positive in 80.4% of PD, 24.2% of post-COVID, and 11.9% of HC participants (*p* < 0.001). PD differed significantly from both post-COVID and HC (both *p* < 0.001), whereas the difference between post-COVID and HC was not significant.

**Table 1:** Demographic and OM-SAA results across study groups

	HC (n = 42)	COVID-19 (n = 95)	PD (n = 51)	*p* value*
Sex (m:f)^a^	20:22	47:48	32:19	0.24
Age^b^	44.8 ± 12.6	48.6 ± 17.6	69.4 ± 9.4	< 0.001 (0.43/< 0.001/< 0.001)
OM-SAA positivity (%)^a^	5 (11.9)	23 (24.2)	41 (80.4)	< 0.001 (0.10/< 0.001/< 0.001)

The significance level was set at *p* < 0.05. Omnibus *p* values are unadjusted. Pairwise post-hoc *p* values were adjusted for multiple comparisons using the Benjamini–Hochberg false discovery rate (FDR) correction for chi-square tests and the Šidák correction for ANOVA

^a^Chi-square test

^b^univariate one-way analysis of variance, ANOVA

*p value for overall group comparison (HC vs COVID-19/HC vs PD/COVID-19 vs PD)

### OM-SAA outcomes in healthy controls and post-COVID-19 participants (Table 2)

**Table 2:** OM-SAA results in the post-COVID cohorts and healthy controls

	HC (n = 42)	COVID-OD (n = 53)	COVID+OD (n = 42)	*p* value*
Sex (m:f)^a^	20:22	25:28	22:20	0.86
Age^b^	44.8 ± 12.6	42.4 ± 15.5	56.3 ± 17.1	< 0.001 (0.83/0.002/< 0.001)
OM-SAA positivity (%)^a^	5 (11.9)	8 (15.1)	15 (35.7)	0.012 (0.65/0.030/0.030)
	Logistic regression models (adjusted for age and sex)			
		β (SE)	Adjusted OR (95%CI)	
	Overall model^c^			
	COVID+OD			
	vs. HC	1.4 (0.6)	3.9, 95% CI 1.2–12.8)	0.047
	vs. COVID-OD	1.1 (0.5)	3.0 (1.0–8.6)	0.047
	Pairwise models			
	COVID+OD			
	vs. HC	1.3 (0.6)	3.7, 95% CI 1.1–12.4	0.046
	vs. COVID-OD	1.1 (0.6)	3.1, 95% CI 1.0–9.1)	0.046

The significance level was set at *p* < 0.05. Post-hoc *p* values were adjusted using the Benjamini–Hochberg false discovery rate (FDR) correction for chi-square and logistic regression analyses and the Šidák correction for ANOVA/ANCOVA. Reported post-hoc *p* values are adjusted; omnibus *p* values are unadjusted

^a^Chi-square test

^b^Univariate one-way analysis of variance, ANOVA

^c^Overall multivariable logistic regression adjusted for age and sex. Neither age nor sex was significantly associated with OM-SAA positivity

^d^Separate pairwise logistic regression models adjusted for age and sex. Benjamini–Hochberg false discovery rate correction was applied to the two pairwise comparisons. Neither age nor sex was significantly associated with OM-SAA positivity

*p value for overall group comparison (HC vs COVID-OD/HC vs COVID+OD/COVID-OD vs COVID+OD)

Table 2 summarizes demographic data as well as OM-SAA results between HC and the post-COVID-19 groups. Age was comparable between the OM-SAA positive and OM-SAA negative groups (mean ± SD: 51.4 ± 17.7 vs. 46.4 ± 15.8 years, *p* = 0.15).

Across the 137 individuals from the HC and post-COVID-19 cohorts, OM-SAA positivity was detected in 20.4% of cases (28/137). OM-SAA positivity rates differed across groups, with 11.9% in HC, 35.7% in COVID+OD, and 15.1% in COVID-OD (Table 2). A chi-square test of independence demonstrated a significant overall association between diagnostic group and OM-SAA status (χ^2^(2)  = 8.84, *p* = 0.012), corresponding to a small-to-medium effect (Cramer’s V = 0.25). Pairwise comparisons with Benjamini–Hochberg FDR adjustment showed that OM-SAA positivity was significantly higher in the COVID+OD group than in both the HC group (FDR-adjusted *p* = 0.030) and the COVID-OD group (FDR-adjusted *p* = 0.030), whereas no significant difference was observed between the COVID-OD and HC group (FDR-adjusted *p* = 0.65). Post-hoc column-proportion tests yielded the same pattern, with significantly higher OM-SAA positivity exclusively in the COVID+OD group, while HC and COVID-OD did not differ.

Moreover, in the overall age- and sex-adjusted logistic regression model, diagnostic group remained significantly associated with OM-SAA positivity after adjustment for age and sex (omnibus χ^2^(4)  = 10.14, *p* = 0.038, Nagelkerke R^2^ = 0.11; Table 2). Group allocation was the only significant predictor, whereas neither age nor sex was associated with OM-SAA positivity.

Within this model, the COVID+OD group showed significantly higher odds of OM-SAA positivity than both healthy controls and the COVID−OD group, whereas no significant difference was observed between the COVID−OD and HC groups (Table 2). Additional age- and sex-adjusted pairwise logistic regression analyses confirmed significantly higher odds of OM-SAA positivity in the COVID+OD group compared with both healthy controls and the COVID−OD group (Table 2). These findings are consistent with the categorical analyses, indicating that elevated OM-SAA positivity is specific to participants with post-COVID-19 olfactory dysfunction.

### Clinical outcomes in healthy controls and post-COVID-19 participants (Table 3)

**Table 3:** Demographic and clinical variables in the post-COVID cohorts and healthy controls

	HC	COVID−OD	COVID+OD	*p*-value*
Whole cohort				
N (Sex; m:f)^a^	42 (20:22)	53 (25:28)	42 (22:20)	0.86
Age, years	44.8 ± 12.6	42.4 ± 15.5	56.3 ± 17.1	< 0.001 (0.83/0.002/< 0.001)
Months since COVID-19^c^	NA	17.2 ± 10.4	17.2 ± 9.3	0.99
SSD^d^	14.8 ± 1.2 (15; 14.5–15.2)	13.8 ± 1.1 (13; 13.5–14.1)	10.4 ± 2.6 (12; 9.6–11.2)	< 0.001 (0.013; < 0.001; < 0.001)
SSI^d^	14.8 ± 1.0 (15; 14.4–15.1)	14.8 ± 1.1 (15; 14.5–15.1)	8.5 ± 1.8 (9; 7.9–9.0)	< 0.001 (1.00; < 0.001; < 0.001)
MDS-UPDRS I^d^	0.5 ± 1.1 (0; 0.2–0.8)	1.5 ± 2.7 (0; 0.7–2.2)	3.0 ± 4.0 (2; 1.7–4.2)	0.008 (0.19; 0.006; 0.34)
MDS-UPDRS II^d^	0.0 ± 0.0 (0; 0.0–0.0)	0.1 ± 0.4 (0; 0.0–0.2)	0.8 ± 2.0 (0; 0.2–1.4)	0.020 (0.90; 0.021; 0.08)
MDS-UPDRS III^d^	0.6 ± 1.6 (0; 0.1–1.1)	0.8 ± 1.6 (0; 0.3–1.2)	2.7 ± 2.6 (3; 1.9–3.5)	0.002 (0.65; 0.002; 0.025)
MDS-UPDRS total^d^	1.1 ± 2.0 (0; 0.5–1.7)	2.4 ± 3.5 (0; 1.4–3.3)	6.5 ± 6.4 (5; 4.5–8.5)	< 0.001 (0.16; < 0.001; 0.015)
MoCA^d^	29.3 ± 0.9 (30; 29.1–29.6)	28.9 ± 1.5 (30; 28.5–29.4)	28.0 ± 1.8 (28; 27.5–28.6)	0.033 (0.16; 0.038; 0.81)
RBD-I score^d^	0.1 ± 0.3 (0; 0.0–0.2)	0.2 ± 0.6 (0; 0.0–0.3)	0.4 ± 0.9 (0; 0.1–0.6)	0.45
Age- and sex-balanced cohorts				
N (Sex; m:f)^a^	33 (17:16)	33 (16:17)	33 (15:18)	0.89
Age, years	49.0 ± 11.0	49.0 ± 11.8	50.9 ± 15.2	0.78
Months since COVID-19^c^	NA	15.1 ± 7.8	16.6 ± 9.5	0.49
SSD^d^	14.9 ± 1.3 (15; 14.5–15.4)	13.7 ± 1.0 (13; 13.3–14.0)	10.2 ± 2.8 (12; 9.2–11.2)	< 0.001 (0.025; < 0.001; < 0.001)
SSI^d^	14.6 ± 1.0 (15; 14.2–15.0)	14.6 ± 1.1 (15; 14.2–15.0)	9.0 ± 1.6 (9; 8.4–9.5)	< 0.001 (1.00; < 0.001; < 0.001)
MDS-UPDRS I^d^	0.6 ± 1.1 (0; 0.2–1.0)	1.8 ± 2.9 (0; 0.7–2.8)	2.8 ± 3.6 (2; 1.5–4.0)	0.006 (0.20; 0.005; 0.39)
MDS-UPDRS II^d^	0.0 ± 0.0 (0; 0.0–0.0)	0.2 ± 0.5 (0; 0.0–0.3)	0.7 ± 2.1 (0; 0.0–1.5)	0.06
MDS-UPDRS III^d^	0.8 ± 1.7 (0; 0.1–1.4)	1.2 ± 1.9 (0; 0.5–1.9)	2.2 ± 2.4 (1; 1.3–3.0)	0.019 (0.74; 0.018; 0.17)
MDS-UPDRS total^d^	1.3 ± 2.1 (0; 0.6–2.1)	3.1 ± 3.9 (1; 1.7–4.5)	5.7 ± 6.1 (5; 3.5–7.8)	< 0.001 (0.20; < 0.001; 0.07)
MoCA^d^	29.2 ± 0.9 (29; 28.9–29.5)	28.8 ± 1.4 (29; 28.3–29.3)	28.0 ± 1.9 (28; 27.3–28.7)	0.001 (0.31; < 0.001; 0.08)
RBD-I score^d^	0.1 ± 0.3 (0; 0.0–0.2)	0.2 ± 0.6 (0; 0.0–0.5)	0.2 ± 0.4 (0; 0.0–0.3)	0.53

The significance level was set at *p* < 0.05. Omnibus *p*-values are unadjusted. Pairwise post-hoc *p*-values were adjusted for multiple comparisons using the Benjamini–Hochberg false discovery rate (FDR) correction for chi-square tests and the Šidák correction for ANOVA/ANCOVA. Data are presented as mean ± standard deviation (median; 95% confidence interval of the mean)

*COVID-OD* post-covid cohort without persistent olfactory dysfunction, *COVID*+*OD* post-COVID cohort with persistent olfactory dysfunction, *HC* healthy controls, *MDS-UPDRS* Movement Disorders Society Unified Disease Parkinson’s Rating Scale, *MoCA* Montreal Cognitive Assessment, *n* number, *OM-SAA* olfactory mucosa seeding amplification assay, *RBD-I* Innsbruck RBD Inventory, *SD* standard deviation, *SSD* Sniffin’ Sticks Discrimination test, *SSI* Sniffin’ Sticks Identification test

^a^Chi-square test

^b^Univariate one-way analysis of variance, ANOVA

^c^Independent two-sample t-test

^d^Univariate analysis of covariance (ANCOVA) corrected for age and sex

^*^p-value for overall group comparison (HC versus COVID-OD/HC vs COVID+OD/COVID-OD versus COVID+OD)

To assess group differences in continuous clinical variables between healthy controls and the post-COVID groups, univariate ANCOVAs adjusted for age and sex were performed (Table 3). MoCA scores differed significantly between groups (F = 3.50, *p* = 0.033, partial η^2^ = 0.05). Age was strongly associated with MoCA performance (F = 42.14, *p* < 0.001, partial η^2^ = 0.24), whereas sex showed no significant effect (F = 3.71, *p* = 0.06). Post-hoc comparisons indicated that this effect was driven by lower MoCA scores in the COVID+OD compared to the HC group (*p* = 0.038). Similarly, MDS-UPDRS total scores also differed significantly between groups (F = 10.27, *p* < 0.001, partial η^2^ = 0.13). Age was significantly associated with MDS-UPDRS total scores (F = 29.21, *p* < 0.001, partial η^2^ = 0.18), whereas sex showed no significant effect (F = 0.63, *p* = 0.43). Post-hoc comparisons revealed higher MDS-UPDRS-total scores in the COVID+OD than in both healthy controls (*p* < 0.001) and the COVID-OD group (*p* = 0.015). Significant group effects were also observed for MDS-UPDRS Part I (F = 5.01, *p* = 0.008, partial η^2^ = 0.07), Part II (F = 4.05, *p* = 0.020, partial η^2^ = 0.06), and Part III (F = 6.63, *p* = 0.002, partial η^2^ = 0.09). Compared with healthy controls, the COVID+OD group had higher scores in Parts I (*p* = 0.006), II (*p* = 0.021), and III (*p* = 0.002). MDS-UPDRS Part III scores were also higher in the COVID+OD group than in the COVID−OD group (*p* = 0.025).

To assess the robustness of the clinical findings despite the age differences between the HC and post-COVID groups, an additional age- and sex-balanced sensitivity analysis was performed including 33 participants per group (see Table 3). The results closely mirrored the primary analyses, confirming significantly lower MoCA scores and higher MDS-UPDRS total scores in the COVID+OD group compared with healthy controls. Similarly, significantly higher MDS-UPDRS Parts I and III scores were observed, whereas the group effect for MDS-UPDRS Part II did not reach statistical significance.

### Clinical outcomes by OM-SAA positivity in post-COVID participants (Table 4)

**Table 4:** Post-COVID participants with versus without positive OM SAA activity

	aSyn-SAA negative (n = 72)	aSyn-SAA positive (n = 23)	*p* value
Sex (m:f)^a^	32:40	15:8	0.13
age^b^	47.3 ± 17.2	52.7 ± 18.5	0.19
SSD^c^	12.8 ± 2.1 (13; 12.3–13.3)	10.7 ± 3.3 (11; 9.3–12.1)	< 0.001
SSI^c^	12.6 ± 3.2 (14; 11.8–13.3)	10.1 ± 3.7 (9; 8.5–11.7)	0.007
MDS-UPDRS I^c^	1.8 ± 3.2 (0; 1.0–2.5)	3.3 ± 3.9 (2; 1.6–4.9)	0.20
MDS-UPDRS II^c^	0.4 ± 1.5 (0; 0.1–0.8)	0.5 ± 1.0 (0; 0.0–0.9)	0.73
MDS-UPDRS III^c^	1.5 ± 2.2 (0; 0.9–2.0)	2.2 ± 2.6 (1; 1.1–3.3)	0.32
MDS-UPDRS total^c^	3.6 ± 5.2 (1.5; 2.4–4.9)	6.0 ± 5.7 (6; 3.5–8.4)	0.24
MoCA^c^	28.8 ± 1.5 (29; 28.4–29.1)	27.9 ± 2.1 (28; 27.0–28.8)	0.20
RBD score^c^	0.2 ± 0.5 (0; 0.1–0.3)	0.5 ± 1.1 (0; 0.0–1.0)	0.12

The significance level is set at *p* < 0.05. Data are presented as mean ± standard deviation (median; 95% confidence interval of the mean)

*aSyn* alpha-synuclein; *COVID-OD* post-covid cohort without persistent olfactory dysfunction; *COVID*+*OD* post-COVID cohort with persistent olfactory dysfunction; *HC* healthy controls; *MDS-UPDRS* Movement Disorders Society Unified Disease Parkinson’s Rating Scale; *MoCA* Montreal Cognitive Assessment; *n* number; *OM-SAA* olfactory mucosa seeding amplification assay; *RBD-I* Innsbruck RBD Inventory; *SD* standard deviation; *SSD* Sniffin’ Sticks Discrimination test; *SSI* Sniffin’ Sticks Identification test

^a^Chi-square test (with Yates’ continuity correction)

^b^Univariate one-way analysis of variance, ANOVA

^c^Analysis of covariance (ANCOVA), adjusted for age and sex

Age was comparable between the OM-SAA positive and OM-SAA negative post-COVID groups (Table 4). To test whether OM-SAA positivity was associated with olfactory function, we conducted a univariate ANCOVA with age and sex as covariates. After adjustment, OM-SAA positive participants had significantly lower SSD scores (F = 12.51, *p* < 0.001, partial η^2^ = 0.12) and SSI scores (F = 7.61, *p* = 0.007, partial η^2^ = 0.08). Age was strongly associated with both SSD and SSI (both *p* < 0.001; partial η^2^ = 0.13 and 0.26, respectively), whereas sex showed no significant effect on either performance. As summarized in Table 4, no significant associations were observed between OM-SAA positivity and MDS-UPDRS total or subscores, MoCA, or RBD-I scores.

## Discussion

In this pilot study OM aSyn seeding activity was detected in approximately one-third of individuals with persistent OD after COVID-19, while OM-SAA positivity rates in post-COVID participants without OD were similar to HC (15.1% vs 11.9%) and thus overall consistent with previous studies [5, 36].

As expected, OM-SAA positivity rate was high in participants with PD (80.4%) and well within the upper range of previously reported studies using the OM matrix [5]. Although overall OM-SAA positivity did not differ significantly between the entire post-COVID cohort and HC, the higher rate among participants with persistent OD suggests that SARS-CoV-2–related injury to the olfactory mucosa may trigger local aSyn misfolding in the context of inflammation. Recent studies of olfactory neurons from patients with persistent post-COVID-19 hyposmia have shown ongoing inflammatory changes, while oligomeric α-synuclein and other markers of neurodegeneration were not increased compared with healthy controls [32]. Whether such peripheral aSyn aggregation can persist and propagate centrally and eventually cause clinically manifest PD or other synucleinopathy remains speculative.

Moreover, COVID+OD participants showed lower MoCA and higher MDS-UPDRS total scores than healthy controls. Differences regarding the latter were primarily driven by higher scores in MDS-UPDRS Part I, reflecting greater impairment in non-motor experiences of daily living, but also by significantly higher MDS-UPDRS Part III scores, indicating subtle objective motor abnormalities. While causality cannot be inferred from the present data, the observed pattern is compatible with previous reports suggesting that persistent post-COVID olfactory dysfunction may co-occur with broader neuropsychiatric sequelae, including fatigue, affective symptoms, sleep disturbances, psychomotor slowing, and cognitive impairment [1, 24, 30, 38, 41].

The finding of higher MDS-UPDRS Part III scores is of particular interest because hyposmia is one of the strongest prodromal markers of Parkinson's disease. Although the absolute motor scores remained low and do not suggest clinically manifest parkinsonism, previous studies have shown that even neurologically healthy individuals with objectively confirmed hyposmia exhibit more subtle motor abnormalities and other prodromal features of Parkinson's disease than normosmic individuals [3, 19, 26, 33]. In parallel, longitudinal studies have demonstrated that a considerable proportion of individuals with idiopathic hyposmia already show evidence of underlying α-synuclein pathology and, in some cases, nigrostriatal dopaminergic dysfunction years before the onset of overt motor symptoms [34]. Together, these observations raise the possibility that persistent olfactory dysfunction may be associated with subtle motor alterations irrespective of the underlying etiology. However, whether the higher MDS-UPDRS Part III scores observed in our cohort reflect persistent post-COVID sequelae, pre-existing prodromal neurodegenerative changes, or an interaction between both mechanisms remains unclear. Notably, OM-SAA positivity was not associated with motor performance in the present study, arguing against a simple relationship between detectable olfactory mucosal α-synuclein seeding and the observed motor findings. Longitudinal studies incorporating dopaminergic imaging together with fluid and tissue biomarkers will be required to determine the biological basis and clinical significance of these subtle motor abnormalities.

Experimental studies have shown detrimental effects of SARS-CoV-2 on substantia nigra dopaminergic neurons as well as stem cell derived dopaminergic neurons [17, 25, 40], and SARS-CoV-2 induced neuroinflammation has been suggested to trigger aSyn misfolding and aggregation with a potential to aggravate or induce a-synucleinopathies [6, 18, 21].

Although the observation of increased rates of OM-SAA positivity in post-COVID-19 subjects with persistent OD seems biologically plausible given SARS-CoV-2 olfactory tropism and the pro-aggregatory potential of neuroinflammation [2, 12, 39], the present results are insufficient to conclude on the prognostic relevance of this finding*.* Longterm follow-up will be needed to shed light on this evidence and to determine whether post-infectious OM-SAA positivity represents a transient observation or a persistent finding. Nevertheless, the enrichment of α-synuclein pathology among post-COVID-19 individuals with hyposmia parallels observations of higher SAA positivity in PD patients with olfactory impairment, further supporting a link between α-synuclein seeding activity and olfactory pathway involvement [35].

While the assessment of OM-SAA positivity in individuals with a history of SARS-CoV-2 infection together with objective olfactory and clinical phenotyping represents a strength of this study, several limitations should be acknowledged. These include the relatively small sample size of post-COVID subjects, the difficulty of recruiting COVID-naïve HC due to the increasing prevalence of SARS-CoV-2 exposure and previous infections in the general population over time, and the cross-sectional design. Because participants were recruited consecutively, age was not fully balanced between the HC and post-COVID groups. To address this potential source of bias, in addition to adjusting the group comparisons for age and sex, we performed a separate age- and sex-balanced sensitivity analysis, which largely confirmed the robustness of the main findings. In particular, the lower MoCA scores and higher MDS-UPDRS total scores observed in the COVID+OD group remained significant. Although the significance of individual MDS-UPDRS subscores differed somewhat in the smaller balanced cohort, the overall pattern and interpretation of the findings remained unchanged. A corresponding sensitivity analysis was not considered necessary for comparisons between OM-SAA-positive and OM-SAA-negative participants, as these groups were more closely balanced with respect to age and all analyses were adjusted for both age and sex.

Furthermore, we cannot fully exclude that some HC may have had an unrecognized (‘silent’) prior SARS-CoV-2 infection, since nucleocapsid antibody testing, which would allow discrimination between infection- and vaccination-induced seroreactivity [10] was introduced at our institution only after HC recruitment had been completed.

## Conclusions

OM-SAA positivity was enriched among individuals with persistent post-COVID OD and correlated with olfactory impairment. Whether this finding reflects a transient post-infectious phenomenon or an early synucleinopathy signature remains to be determined.

## Data Availability

De-identified data supporting the findings of this study will be made available on Zenodo at https://doi.org/10.5281/zenodo.23349375.

## Declarations

### Trial registration number

ClinicalTrials.gov: NCT05401773.
